# Distinct microbial assemblages associated with genetic selection for high- and low- muscle yield in rainbow trout

**DOI:** 10.1186/s12864-020-07204-7

**Published:** 2020-11-23

**Authors:** Pratima Chapagain, Donald Walker, Tim Leeds, Beth M. Cleveland, Mohamed Salem

**Affiliations:** 1 Department of Biology and Molecular Biosciences Program, Middle Tennessee State University, Murfreesboro, TN 37132 USA; 2 National Center for Cool and Cold-Water Aquaculture, ARS-USDA, Kearneysville, WV 25430 USA; 3 Department of Animal and Avian Sciences, University of Maryland, College Park, MD 20742-231 USA

**Keywords:** Aquaculture, Gut microbe function, Microbiota, Selective breeding, Muscle yield, Fillet, ARS-FY-H, ARS-FY-L

## Abstract

**Background:**

Fish gut microbial assemblages play a crucial role in the growth rate, metabolism, and immunity of the host. We hypothesized that the gut microbiota of rainbow trout was correlated with breeding program based genetic selection for muscle yield. To test this hypothesis, fecal samples from 19 fish representing an F2 high-muscle genetic line (ARS-FY-H) and 20 fish representing an F1 low-muscle yield genetic line (ARS-FY-L) were chosen for microbiota profiling using the 16S rRNA gene. Significant differences in microbial assemblages between these two genetic lines might represent the effect of host genetic selection in structuring the gut microbiota of the host.

**Results:**

Tukey’s transformed inverse Simpson indices indicated that high muscle yield genetic line (ARS-FY-H) samples have higher microbial diversity compared to those of the low muscle yield genetic line (ARS-FY-L) (LMM, χ2(1) =14.11, *p* < 0.05). The fecal samples showed statistically distinct structure in microbial assemblages between the genetic lines (F_1,36_ = 4.7, p < 0.05, R^2^ = 11.9%). Functional profiling of bacterial operational taxonomic units predicted characteristic functional capabilities of the microbial communities in the high (ARS-FY-H) and low (ARS-FY-L) muscle yield genetic line samples.

**Conclusion:**

The significant differences of the microbial assemblages between high (ARS-FY-H) and low (ARS-FY-L) muscle yield genetic lines indicate a possible effect of genetic selection on the microbial diversity of the host. The functional composition of taxa demonstrates a correlation between bacteria and improving the muscle accretion in the host, probably, by producing various metabolites and enzymes that might aid in digestion. Further research is required to elucidate the mechanisms involved in shaping the microbial community through host genetic selection.

**Supplementary Information:**

The online version contains supplementary material available at 10.1186/s12864-020-07204-7.

## Background

Aquaculture is one of the fastest-growing industries and plays a vital role in fulfilling the global requirements for human protein consumption [1]. Growth rate and muscle yield are key traits affecting the profitability of aquaculture. Understanding the mechanisms for fast and efficient muscle growth is beneficial for developing strategies that improve these characteristics. Muscle growth in farmed fish is influenced by host genetics and factors such as nutrition and environmental condition [2]. Traditional phenotype-based genetic selection is used to improve fish production traits. However, it is not possible to apply this muscle-yield selection strategy on potential breeding candidates since measuring this trait requires sacrificing the fish prior to sexual maturation [3]. Family-based selection procedures have been undertaken by the United States Department of Agriculture at the National Center for Cool and Cold Water Aquaculture (NCCCWA) to improve growth rate and muscle yield in rainbow trout. The 5th-generation fast-growth line families were the base population for the fillet yield selection lines [4]. A family-based selection for muscle yield in a closed, pedigreed population was used to develop high- muscle yield (ARS-FY-H), randomly mated control (ARS-FY-C), and low- muscle yield (ARS-FY-L) genetic lines.

The gastrointestinal compartments of fish contain large microbial communities that play an essential role in homeostasis, physiology, and gut development [5–7]. Microbiota residing in the host gut act as a barrier for the colonization of pathogenic bacteria [8]. These bacteria can produce vitamins B and K, short-chain fatty acid, butyric acid, and different antimicrobial metabolites, which may improve the host growth rate and muscle percentage [9]. Host genetics also play a crucial role in shaping the gut microbiome [10]. In addition to host genotype, diet alteration (plant- and animal-based meal) can change the population of the host microbiota as fish subsequently obtain their microbiota from the first-feed they eat [11, 12]. In humans, previous studies showed an influence of gut microbiota on muscle fitness and degradation [13, 14]. Symbiotic microbial communities residing in humans supply short-chain fatty acids (SCFAs) to the skeletal muscle resulting in improved muscle percentage and fitness, whereas, dysbiosis (imbalance in microbiota) results in muscle degradation due to increased intestinal permeability and liberation of endotoxin into circulation [14–16]. Muscle constitutes about 50–60% of the fish body weight [17] and plays a significant role in the regulation of nutrient metabolism, growth, and inflammation in humans and fish [18–20]. Similarly, Lahiri et al. reported a correlation between the gut microbiota and the skeletal muscle mass in mice. Mice lacking gut microbiota showed muscle atrophy, decreased expression of insulin-like growth factor 1, and reduced transcription of genes associated with skeletal muscle growth and mitochondrial function [21], suggesting a potential role of the gut microbiota in improving muscle yield and reducing muscle atrophy.

Microbiota transmit nutrient signals to their hosts, which might shape the gut microbiome in every stage of life based on diet intake, behavioral change, and environmental influence [22]. Research had shown that transplantation of gut microbes in an animal may improve the muscle mass percentage, function, and reduction in muscle atrophy markers [21]. Few studies have investigated the correlation of gut microbial composition in muscle development and metabolic profile in fish. Therefore, the overall objective of our research was to study the gut microbiota in two genetic lines of rainbow trout selected for high- and low-muscle yield. We postulated that microbial diversity is associated with genetic selection for improved muscle yield.

## Results and discussion

**Fig. 1:**
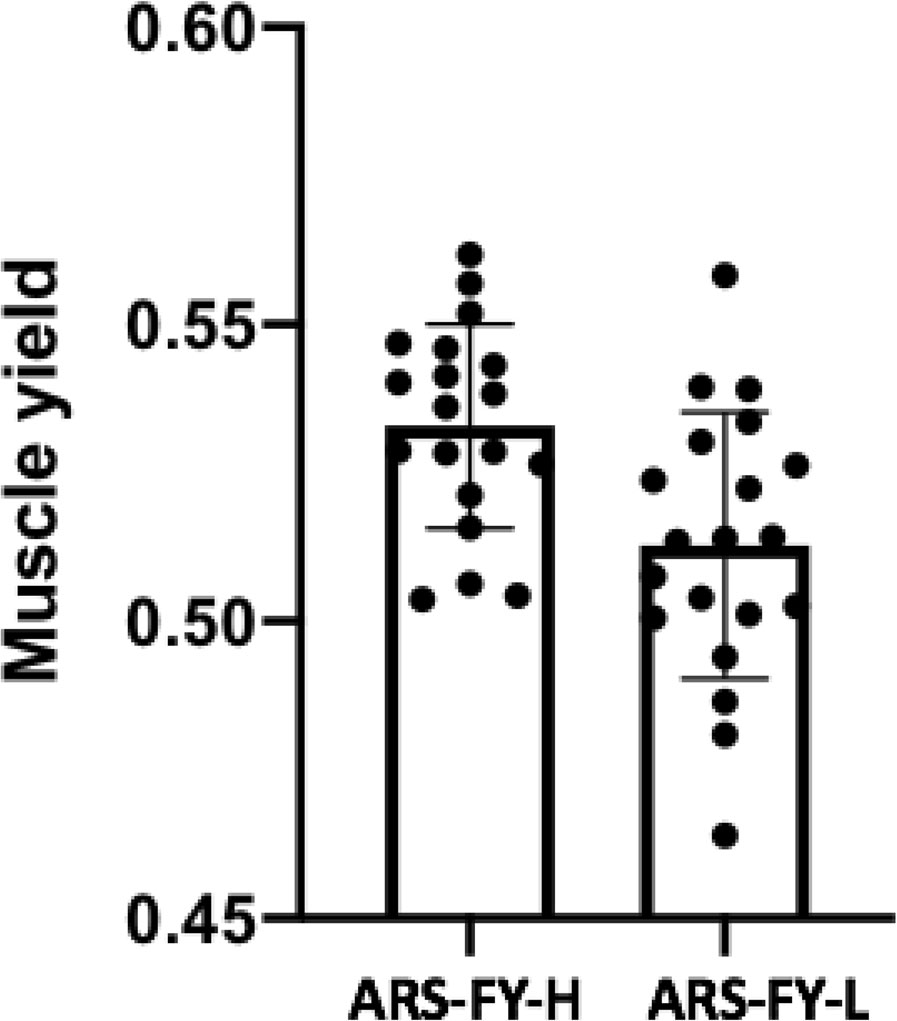
Significant differences in the muscle yield among samples collected from the ARS-FY-H (high yield) and ARS-FY-L (low yield) rainbow trout genetic lines. The statistical significance of the differences in muscle yield between the two groups was tested by a one-way Mann-Whitney U test (*p* = 0.0029)

Divergent selection was practiced for fillet yield to develop high (ARS-FY-H) and low (ARS-FY-L) yield genetic lines of rainbow trout. The two fish groups used in this study were collected after two generations of selection and were statistically different in their average muscle yield as indicated by a one-way Mann-Whitney U test (*p* < 0.05; Fig. 1). The mean muscle yield of the high (ARS-FY-H) genetic was 0.53 ± 0.01%, and that of the low (ARS-FY-L) genetic line was 0.51 ± 0.02%.

### Comparison of gut assemblages in high-(ARS-FY-H) and low-(ARS-FY-L) muscle yield genetic lines

**Fig. 2:**
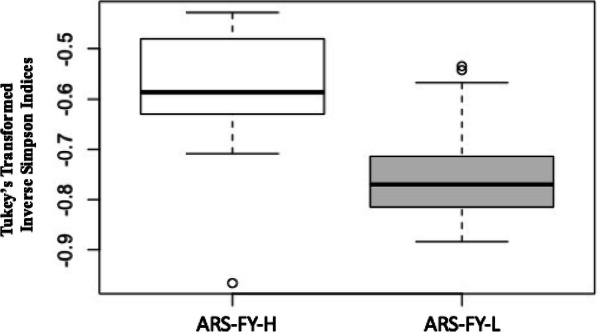
Alpha diversity of ARS-FY-H (high) and ARS-FY-L (low) genetic lines. The box plots indicate higher microbial diversity in the ARS-FY-H samples (*p* < 0.05). Boxplots show the median value as a bold black bar, the upper and lower limits of the box being the third and first quartile of the data, the whiskers extend up to 1.5 times the interquartile range, and open circles are outlier points

**Fig. 3:**
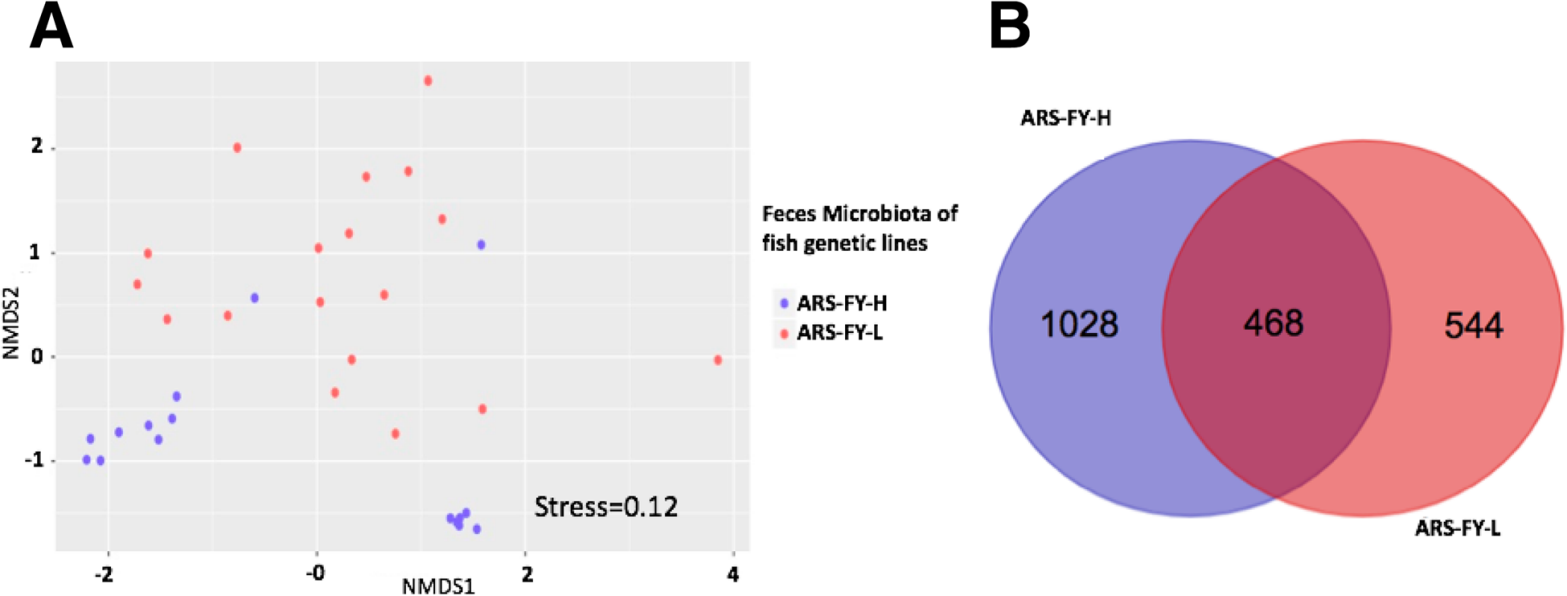
**A**. nMDS ordination of microbial assemblage structure in the ARS-FY-H (high) and ARS-FY-L (low) genetic lines. **B.** Venn-diagram showing numbers of shared and unique OTUs between the two genetic lines

Fish were reared and harvested under identical conditions, however, there was a significant difference in gut microbes between the two harvest days in the high- (ARS-FY-H; F_1,15_ = 8.24, *p* < 0.05, R^2^ = 37.06%) but not low-muscle yield genetic lines (ARS-FY-L; F_1,17_ = 0.85, *p* > 0.05). Therefore, harvest day was treated as a random effect in all models to test for the main effect of genetic line. Using a linear mixed model, we tested for differences in gut alpha diversity between fish genetic lines and found that diversity was greater in high (ARS-FY-H) genetic line (LMM, χ2(1) = 14.11, *p* < 0.05, Fig. 2) when controlling for the harvest day effect. Both nMDS ordination and PERMANOVA results (F_1,36_ = 4.7, p < 0.05, R^2^ = 11.9%) indicated that the muscle-yield genetic line was predictive of gut microbial assemblages in rainbow trout (Fig. 3a). There were no significant differences in multivariate dispersion between gut assemblages of low (ARS-FY-L) and high (ARS-FY-H) genetic line samples. A total of 468 OTUs were shared between the two genetic lines (Fig. 3b). The high (ARS-FY-H) muscle-yield genetic line samples had almost double the number of unique OTUs compared to the low (ARS-FY-L) muscle-yield genetic line.

Together, these results indicate that the muscle-yield genetic lines are predictive of gut microbial assemblages and suggest that host genetic selective breeding might help curate a particular gut microbial assemblage. This notion is supported by recent studies in tilapia, showing host genetic selection for cold thermal tolerance has an effect on the microbiome [23]. Similarly, studies in stickleback fish identified an association between gut microbial differences and host genetic divergence [24]. Previous work from our lab group revealed significant variation in beta diversity of the bacterial communities of rainbow trout families showing variation in growth rate [25]. Together, these studies indicate a substantial impact of host selection or genetics in predicting host-associated microbial assemblages.

### Taxonomy and functional diversity correlate with selection for fish muscle yield

**Fig. 4:**
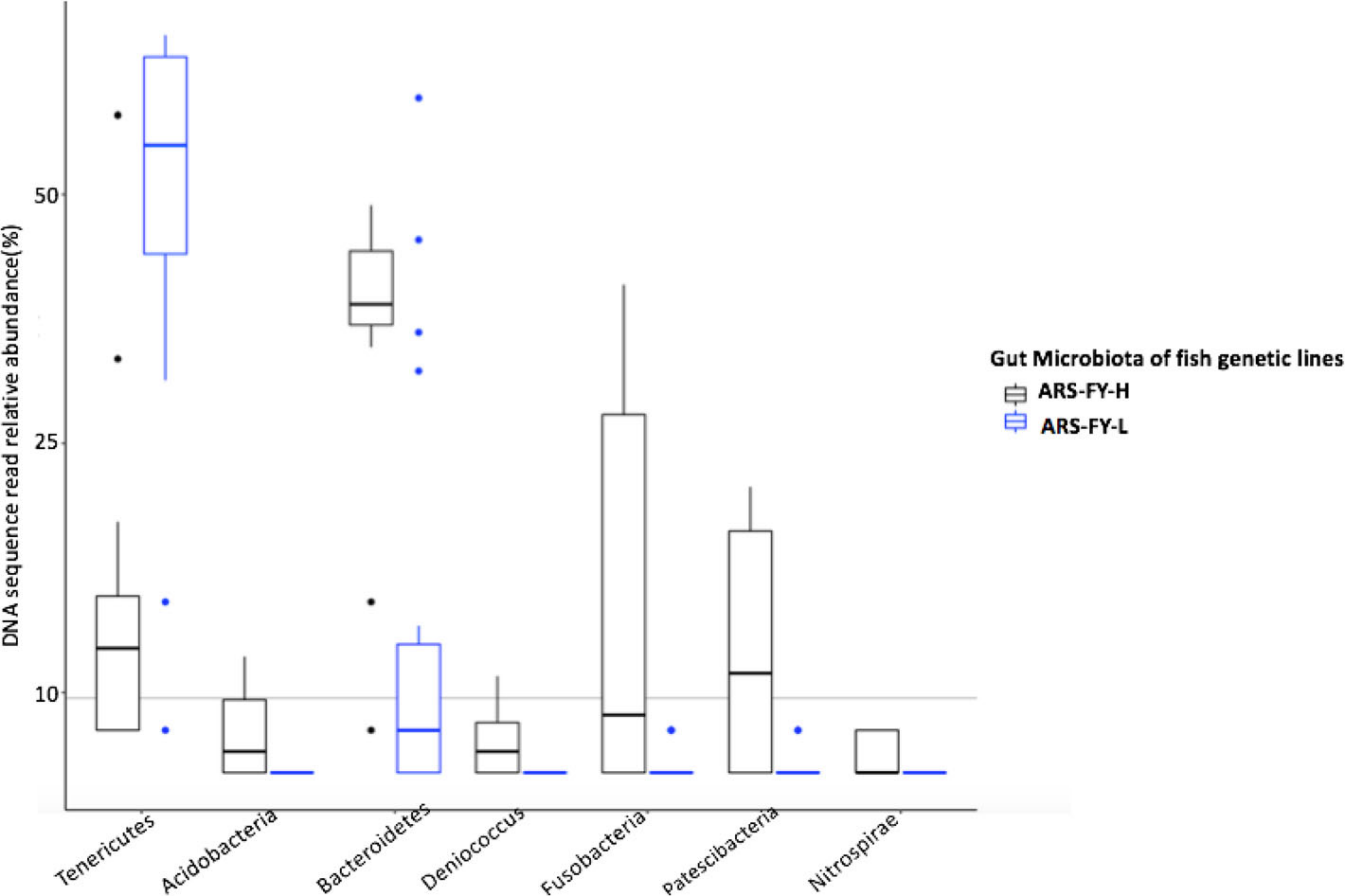
Comparison of the bacterial phyla with > 5% abundances between the ARS-FY-H (high) and ARS-FY-L (low) genetic lines. Boxplots show the median value as a bold black bar, the upper and lower limits of the box being the third and first quartile of the data, the whiskers extend up to 1.5 times the interquartile range, and the closed colored circles are outlier points

A total of eight phyla, 12 classes, 36 families, and 64 genera had significant differences in abundance between the two genetic lines (Kruskal-Wallis test; *p* < 0.05, Additional file 1). Phyla Bacteroidetes, Fusobacteria, Deniococcus, Acidobacteria, Patescibacteria, and Nitrospora had higher abundance in the high (ARS-FY-H) muscle yield genetic line, whereas, the phylum Tenericutes had higher relative abundance in the low (ARS-FY-L) muscle yield genetic line (Fig. 4). Using a genus-level comparison, some unclassified genera belonging to family Burkholderiaceae and Gammaproteobacteria had higher abundance in high (ARS-FY-H) muscle yield genetic line. The genera *Bacteroides*, *Deniococcus, Lutelibacter, Nitrosomonas, Pasteurella,* and *Negativibacillus* were present only in the high (ARS-FY-H) muscle yield genetic line.

Higher abundances of the phyla Bacteroidetes, Fusobacteria, Deniococcus might be associated with higher muscle percentage, as these taxa are known symbionts and produce metabolites such as SCFAs that are beneficial to the host [26–29]. For example, genera in the phylum Bacteroidetes are associated with degradation of complex protein polymers and these are responsible for the formation of SCFAs like succinic acid, propionic acid, and acetic acid as the end products [28]. Similarly, genera in the phylum Fusobacteria, a phylum reported to be abundant in freshwater fish guts [30, 31], may produce butyrate, which supplies energy to gastrointestinal cells and inhibits pathogens in freshwater fish [32]. Fusobacteria are known to colonize the gut of zebrafish, synthesize vitamins, excrete butyrate, and metabolites associated with improving fish health [33]. Similarly, bacteria in the phylum Deinococcus can metabolize glucose [34]. Conversely, phylum Tenericutes had higher abundance in the low (ARS-FY-L) muscle yield genetic line samples. This phylum is found in the gut of Fathead minnows [35], however, the functional role of this phylum is not well studied in fish. A study on crabs showed that the Tenericutes phylum is correlated with Hepatopancreatic necrosis disease [36].

There are 64 taxonomic groups with significant differential abundances between the two muscle yield lines. Among them, 21 groups have > 10% and 4 had > 15% differential abundances. These 21 taxonomic groups include *Arcicella, *unclassified Clostridiaceae*, *unclassified Burkholderiaceae*, Pedobacter, *Absconditabacteriales*, Arsenicibacter, *Chiinophagaceae*, Cloacibacterium, Hydrogenophaga, Rhizorhapis, Rhodoferax, *unclassified Sphingomonadaceae*, Sphingorhabdus, *unclassified Spirosomaceae*, Thermomonas, Thiothrix, Undibacterium, *unclassified Veillonellaceae*, Chryseobacterium.* All of these taxonomic groups have greater abundance in the high (ARS-FY-H) muscle yield genetic line, whereas, the genus *Mycoplasma* (with differential abundance 16.8%), and unclassified Firmicutes (with differential abundance 7.9%) have higher abundance in the low (ARS-FY-L) muscle yield genetic line (Additional file 1).

A study in rainbow trout showed that the genus *Clostridium butyricum* in the family Clostridiaceae, enhances the disease resistance in the host against the pathogen *Vibrio* by increasing the phagocytic activity of leucocytes [37]. Inhibiting pathogenic bacteria from colonizing the host might help to improve host health, including growth rate and metabolism. In addition, these genera have been used as a probiotic to improve immune response and survival in *Paralichthys olivaceus fish* [38]*. Arcicella,* belonging to phylum Bacteroidetes has been identified in freshwater environments, and these bacteria can ferment carbohydrates [39]. Similarly, bacteria from the genus *Pedobacter* is dominant in the gut of healthy Atlantic Salmon [40].

The genus *Bacteroides* is an important group of bacteria colonizing the intestine of a wide variety of hosts, including humans [41, 42], mice [43], and tilapia fish [44]. These bacteria ferment carbohydrates and produce short-chain fatty acids (SCFAs) like acetate, propionate and butyrate. These SCFAs are key regulators of skeletal muscle metabolism and function [45]. A recent study showed that species of *Cloacibacterium* isolated from the Abalone intestine can hydrolyze starch and ferment sugars like glucose, galactose, fructose, maltose, mannose, and produce fatty acids [46]. Both the *Bacteroides* and *Cloacibacterium *have greater abundance in high (ARS-FY-H) muscle yield genetic lines and are associated with digestion, fatty acid metabolism, pathogen inhibition, which, are linked to host health and digestion. Family Burkholderiaceae had a higher abundance in high (ARS-FY-H), and genus Burkholderia belonging to this family were reported as the most abundant genus in the fish gut [47]. However, their role in muscle yield and or host health in fish has not been reported before.

Taxa with significantly higher abundance in the low (ARS-FY-L) muscle yield genetic line were *Mycoplasma* (16.8%) and the unclassified phylum Firmicutes (7.9%). A previous study showed that the genus *Mycoplasma* is the most abundant taxon in adult Atlantic salmon [48]. Bacteria belonging to this genus have also been described as pathogenic in gills of the fish *Tinca tinca* [49]. The lesser abundance of unclassified Firmicutes in the low (ARS-FY-L) muscle yield genetic line might be associated with decreased body weight or correlated with a decrease in muscle percentage in fish. The ratio of Firmicutes to Bacteroides has been shown to correlate with weight gain in humans [50] and this trend could exist in fish as well. A previous study in our laboratory showed that body weight of rainbow trout is moderately correlated with muscle yield, regression coefficient (R2) values of 0.56 [51].

**Fig. 5:**
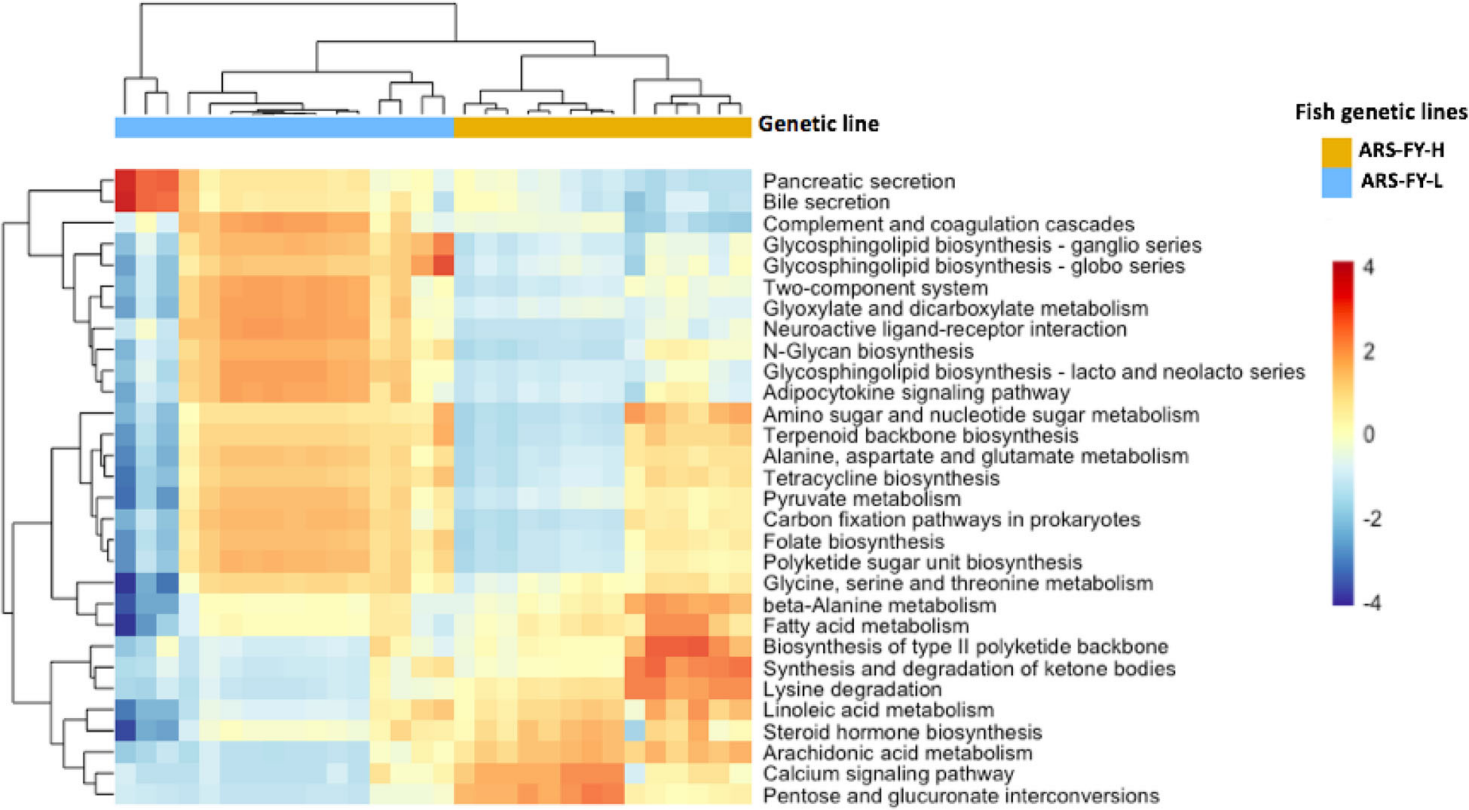
Heatmap showing metabolic pathways that statistically differed between the ARS-FY-H (high) and ARS-FY-L (low) genetic lines. Samples and pathways are clustered based on Euclidean distances. The abundance of each pathway was scaled to a range (− 4, 4) with red and blue colors representing higher and lower pathway abundance, respectively

Tax4Fun analyses were used in this study to enumerate differential functional capabilities of microbial communities in high (ARS-FY-H) and low (ARS-FY-L) genetic lines (Fig. 5). Bacterial functional pathways related to calcium signaling, pentose and glucuronate interconversions, synthesis and degradation of ketone bodies, linoleic acid metabolism, lysine degradation, and arachidonic acid metabolism were enriched in most of the high (ARS-FY-H) genetic line samples. Microbial pathways involved in fatty acid metabolism are known to supply energy to muscle cells, which is essential for muscle growth [52], and was more abundant in the high (ARS-FY-H) genetic line. Genus *Bacteroides* belonging to phylum Bacteroidetes showed higher abundance in the high (ARS-FY-H) genetic line, and is associated with fatty acid metabolism thus producing SCFAs [53]. A study in mice revealed that SCFAs produced by microbes in the gut supported muscle function by preventing muscle atrophy and boosting muscle strength [21].

Similarly, microbe mediated lysine degradation increased production of SCFAs (butyrate and acetate) in the human gut [54]. The genus *Fusobacterium* was more abundant in the high (ARS-FY-H) muscle yield genetic line and known to be involved in lysine degradation and production of SCFAs [55]. Microbial synthesis and degradation of ketone bodies (KB), identified in the ARS-FY-H samples, were reported as associated with increased muscle mass in humans [56, 57]. Ketone bodies make an energy substrate that supplies energy to the brain and muscles, contributing to the maintenance of energy homeostasis through regulation of lipogenesis [56]. Arachidonic acid metabolism is essential for the functions of skeletal muscle and the immune system, which might be associated with increased muscle mass and host health [58, 59]. The family Clostridiaceae had greater abundance in high (ARS-FY-H) muscle yield genetic line fish and was reported as correlated with enriched pentose and glucuronate interconversions [60]. Bacteria associated with this pathway are involved in the breakdown of complex substrates in pig gut microbiomes and improved carbon and energy uptake in the host [60]. 

Fish with low (ARS-FY-L) muscle yield had unique functional profiles that differed from high (ARS-FY-H) muscle yield samples. For example, pyruvate metabolism, amino acid metabolism, folate biosynthesis, glycosphingolipid biosynthesis, glyoxylate and dicarboxylate metabolism, adipocytokine signaling pathway, and two-complement system were enriched in most of the low (ARS-FY-L) genetic line samples (Fig. 5). Glycosphingolipids act as negative regulators of skeletal muscle differentiation and growth in rats [61–63]. Similarly, bile secretion is associated with lipid digestive functions [64, 65] and may reduce adiposity in the host, which might result in lower muscle mass. In spite of the differential enrichment of pathways between the muscle yield groups, further investigation should be done to validate the role of these microbial pathways in the host.

## Conclusion

In this study, the gut microbial assemblage (alpha and beta) diversity correlated with selectively-bred muscle yield genetic lines. Microbial differences between the two genetic lines could be observed as a host genetic selection signature on the gut microbiota. Both differences in taxonomic groups of microbes and their functional predictions correlated with muscle yield. Our results suggest a role of specific microbial taxa in improving host muscle growth and metabolism. 

## Methods

### Fish husbandry and harvest

Fish were produced at the NCCCWA as a part of our ongoing selective breeding program. Briefly, full-sibling families (ARS-FY-H = 99; ARS-FY-L = 23) were produced from single-sire × single-dam mating events. Each family was reared separately from hatch through approximately 30 g (4 months post-hatch) when 8 fish per family were anesthetized (100 mg/L tricaine methanesulfonate, M-222) and uniquely tagged by inserting a passive integrated transponder (Avid Identification Systems Inc., Norco, CA) into the peritoneal cavity. After tagging, fish were comingled and reared in a single, 1800-L tank that also housed contemporary fish (*n* = 118) from the ARS-FY-C line. The tank received identical water from a partial re-use system and water temperature was maintained at approximately 13 °C for the entirety of the grow-out period. Fish were split at random into a total of two 1800-L and two 3800-L tanks as they grew to maintain biomass densities below 100 kg/cubic meter. At approximately 13 months of age, fish used in the current study were split into three replicate 800-L tanks (*n* = 46 fish per tank). One week before harvest, the fish were split at random into four 800-L tanks (34–35 fish per tank) to allow the harvest of two complete tanks on each of two successive days and thus minimize netting-associated stress associated with harvesting of a partial tank. Fish were fed a commercial diet (Finfish G, 42% protein, 16% fat; Ziegler Bros Inc., Gardners, PA) using automatic feeders (Arvotec, Huutokoski, Finland) that provided feed at a daily ration that was considered as slightly below satiation.

At approximately 15 months post-hatch, fish were euthanized using an overdose of anesthetic (300 mg/L MS-222) and processed for analysis of the muscle yield trait. Fish were not fed the day prior to and the day of harvest. Twenty families were pre-selected from the high (ARS-FY-H) and low (ARS-FY-L) genetic lines (40 families total within four tanks; *n* = 1 fish per family) for fecal collection at harvest; selection was based on divergent mid-parent breeding values and to maximize genetic diversity within each line. Due to a mortality of a high (ARS-FY-H) genetic line fish, 39 fecal samples were collected for this study, 19 from the high (ARS-FY-H) muscle yield genetic line (11 and 8 samples from the first and second harvest dates, respectively) and 20 representing the low (ARS-FY-L) genetic line (13 and 7 samples from the first and second harvest dates, respectively). Fecal samples were stripped manually into sterile Eppendorf tubes (Eppendorf, Hauppauge, NY), then stored in a − 80 °C freezer until analysis. Fish were eviscerated and the carcasses were placed on ice and held in a 4 °C refrigerator overnight for analysis of muscle yield the following day.

### DNA extraction, library preparation and sequencing

To extract DNA, fecal samples from 19 high (ARS-FY-H) and 20 low (ARS-FY-L) genetic line fish were subjected to DNA isolation using a Promega Maxwell DNA Isolation Kit (Promega Corporation, Madison, WI), as we previously described [25] with a minor modification where 20 μL of lysozyme was added in samples to facilitate cell wall lysis. Briefly, 200 mg of fecal sample was added to a microtube containing 160 μL of incubation buffer, 20 μL proteinase k solution, and 20 μL lysozyme. The mixture was incubated at 70 °C overnight, and after incubation, 400 μL of lysis buffer was added to the mixture and the sample was vortexed briefly. The samples were then subjected to the Maxwell 16 Automated DNA purification machine and the DNA was collected in a 50-μL elution buffer.

Library preparations and sequencing were done based on 16S rRNA sequencing strategy using the Illumina 16S Metagenomic Sequencing Library Preparation Guide. Briefly, 10 μM of 515F and 10 μM of 806R primers amplifying V4 regions were used to target 16S rRNA gene using McLAB HiFi master mix using polymerase chain reaction (PCR). The final PCR reaction consisted of 12.5 μL 2x HiFi, 1 μL of 10 μM 515F primer and 1 μL of 10 μM 806R primer, 5 μL DNA and 5.5 μL sterile nuclease-free water. The PCR product was then subjected to size selection using a magnetic bead capture kit (Ampure; Agencourt). After the first PCR clean up, dual indexed primers were used to amplify the V4 region as described by Kozich et al. [66]. After indexing, samples were again size selected using a magnetic bead capture kit (Ampure; Agencourt). PCR products were quantified after amplification and indexing using a Qubit fluorometer (Invitrogen, Carlsbard, CA) and fragment size (approximately 450 bp) was visualized on a 1.5% gel electrophoresis stained with SYBR safe, then normalized to 4 nM. Samples were loaded onto an Illumina MiSeq flow cell and sequencing was done using 250 bp-paired end sequencing using a 500 cycle V2 reagent cartridge (Illumina, Inc., San Diego, CA) according to the manufacturer’s instructions [67].

### Bioinformatics analysis

A total of 28,518,046 paired-end raw sequences were obtained during the Miseq run. Sequencing data were analyzed using Mothur (v.1.40.2, www.mothur.org) according to the Mothur Illumina Miseq standard operating procedure (SOP) [66, 68] with several modifications. After forming contigs, the total number of sequences was 11,020,368, and *pcr.seqs* command was used to trim primers and adaptors to the V4 region. The median length of the sequences was determined as 253 by using the *summary.seqs* command [69]. *Screen.seqs* command was used to remove sequences with length > 254 bp and < 251 bp containing homopolymers of > 8, and with ambiguous base calls. The *split.abund* command was used to keep Operational Taxonomic Units (OTUs) with more than two reads [70]. The SILVA v123 database [71] was used to align the sequences and those that failed to align, or classified as Archaea, chloroplast, Eukaryote, mitochondrial, or unknown were excluded from the analysis. Chimeric sequences were detected by *chimera.vsearch* and removed from the analysis. The remaining sequences were clustered using *cluster.split* [72] at a threshold of > 97% sequence similarity. Operational Taxonomic Units with relative abundance < 10 across all samples were removed from the analysis by using the *remove.rare* command [73, 74]. The final data set was subsampled at 2420 sequences to normalize the data set for statistical analyses. DNA extraction and library preparation blanks were included during sequencing and bioinformatics, and all OTUs within these samples were removed from the final analysis. The code used during bioinformatics analysis, the taxonomy file, and the shared sample × OTU matrix are all included in Additional files 2, 3, and 4, respectively.

### Statistical analysis

To test for the statistically significant differences of the muscle yield between the two groups, a one-way Mann-Whitney U test (Prism, GraphPad Software, Inc., La Jolla, CA) was performed. Statistical analyses (alpha diversity, beta diversity, microbial functional profiling pathways) were performed in R version 3.5.2 using the packages *vegan* [75], *plyr* [76], *dplyr* [77], *ggplot2* [78], *lmerTest* [79], *pheatmap*, *MuMIn* [80], *lme4* [81], *Tax4Fun* [82], *DEseq2* [83], *rcompanion* [84], *grid* [85], and *TidyVerse* [86].

### Alpha and Beta diversity analysis of fecal samples between high (ARS-FY-H) and low (ARS-FY-L) muscle yield genetic lines

Sixteen fecal samples (that passed QC during bioinformatics analysis) from the high (ARS-FY-H) and 18 fecal samples (that passed QC) from the low (ARS-FY-L) muscle yield line were used for this analysis. A Tukey’s ladder of power transformation was performed to fit inverse Simpson values to a Gaussian distribution. Alpha diversity between the genetic lines was compared using a linear mixed-effects model (LMMs) with the genetic line as a fixed effect and harvest day set as a random effect (package *lme4*) [87].

Beta diversity was calculated to test if muscle yield genetic line was predictive of the gut microbiota. To do this, a Bray-Curtis dissimilarity matrix was generated using the *vegdist* function in the Vegan package [88]. The *betadisper* function was used to test for the homogeneity of multivariate dispersion between gut assemblages from high (ARS-FY-H) and low (ARS-FY-L) muscle yield genetic lines. The *metaMDS* function in Vegan was used to generate a non-metric multidimensional scaling ordination (nMDS), which was then plotted using *ggplot2* [89]. The *adonis* function in Vegan was used to perform PERMANOVA on Bray-Curtis dissimilarity values to determine if the genetic line was predictive of gut assemblages, while controlling for harvest day effect (harvest day as strata, 999 permutations). An indicator species analysis was performed in Mothur to determine the microbial assemblages that were characteristic of muscle-yield genetic lines [29]. A Kruskal-Wallis test was used to assess differences in relative abundances of taxa between the genetic lines. The nMDS ordination showed a pattern suggesting a ‘harvest day’ based effect; therefore, we subset our samples into two data frames based on independent harvest days. Both data frames had nearly equal numbers of gut microbial samples from the two genetic lines, including 10 samples from high (ARS-FY-H) and 11 samples from low (ARS-FY-L) - in harvest day 1, and 6 from high (ARS-FY-H) and 7 from low (ARS-FY-L) - in harvest day 2. Separate Bray-Curtis dissimilarity matrices were generated for each data frame, followed by nMDS ordination values calculated and plotted in *ggplot2.* PERMANOVA was used to test for differences in microbial assemblages with genetic line set as a fixed effect.

### Beta diversity analysis of the fecal samples, feed and water

Feed and water samples were sequenced to determine whether the fecal microbial assemblages of trout differed from the environment. Beta diversity was calculated based on a Bray-Curtis dissimilarity matrix representing sample-to-sample pairwise distances using the *vegdist* function, and non-metric multidimensional scaling (nMDS) ordination was used for visualization using the *metaMDS* function and plotting in *ggplot2*. The *adonis* function was performed to determine if sample type (gut, feed, water), set as a fixed effect, was descriptive of microbial assemblages. The detailed methodology and results for this experiment have been included in Additional file 5.

### Functional annotation of 16S rRNA sequence data

Phylotype based OTU clustering and classification was performed using the *phylotype* command in Mothur to investigate microbial functional and metabolic capacities of OTUs. The *shared* file was then converted to the *biome* format using the *make.biom* command in Mothur. The Tax4FUN package in R was used to predict the microbial functional and metabolic capacities by linking 16S rRNA gene-based taxonomic profiles to KEGG reference profiles [82]. The normalized KEGG pathway output was used to investigate the enrichment of microbial pathways between the genetic line samples using DESeq2. Informative pathways associated with host-microbiome interactions with an average FTU score of 0.55 and an adjusted *p*-value less than 0.001 were selected for heatmap visualization using the *pheatmap* R package [90]. The R code used during the analysis has been included in Additional file 6, and statistical results for all analyses are included in Additional file 7.

## Supplementary Information

**Additional file 1.** Differentially abundant taxa. Table including taxa having significant differences in abundance between the two genetic lines.

**Additional file 2.** Mothur Standard Operating procedure for microbiota profiling.

**Additional file 3.** Mothur Taxonomy file containing taxa information of all the OTUs.

**Additional file 4.** Mothur Shared file used to calculate alpha and beta diversity metrics.

**Additional file 5.** Comparison of gut and environmental samples.

**Additional file 6.** R code.

**Additional file 7.** Statistical analysis results from alpha and beta diversity analysis.

## Data Availability

All data are provided in additional files.

## Abbreviations

ARS-FY-H: High muscle genetic line
ARS-FY-L: Low muscle genetic line
OTU: Operational Taxonomic Unit
PERMANOVA: Permutation multivariate analysis of variance
KEGG: Kyoto encyclopedia of genes and genomes
nMDS: non-metric Multidimensional Scaling
SCFAs: Short chain fatty acids
KB: Ketone Bodies

## References

[1] Little DC, Newton RW, Beveridge MC (2016). Aquaculture: a rapidly growing and significant source of sustainable food? Status, transitions and potential. Proc Nutr Soc.

[2] Hamre, J., E. Johnsen and K. Hamre. A new model for simulating growth in fish. 2014;PeerJ(2):e244. 10.7717/peerj.244 PMC3912445 24498574

[3] Al-Tobasei R, Ali A, Leeds TD, Liu S, Palti Y, Kenney B, Salem M (2017). Identification of SNPs associated with muscle yield and quality traits using allelic-imbalance analyses of pooled RNA-Seq samples in rainbow trout. BMC Genomics.

[4] Leeds TD, Vallejo RL, Weber GM, Pena DG, Silverstein JS. Response to five generations of selection for growth performance traits in rainbow trout (*Oncorhynchus mykiss*). Aquaculture. 2016;465:341–51.

[5] Rosenbaum M, Knight R, Leibel RL (2015). The gut microbiota in human energy homeostasis and obesity. Trends Endocrinol Metab.

[6] Feng Q, Chen WD, Wang YD (2018). Gut microbiota: an integral moderator in health and disease. Front Microbiol.

[7] Butt RL, Volkoff H (2019). Gut microbiota and energy homeostasis in fish. Front Endocrinol (Lausanne).

[8] Pickard JM, Zeng MY, Caruso R, Nunez G (2017). Gut microbiota: role in pathogen colonization, immune responses, and inflammatory disease. Immunol Rev.

[9] Rowland I, Gibson G, Heinken A, Scott K, Swann J, Thiele I, Tuohy K (2018). Gut microbiota functions: metabolism of nutrients and other food components. Eur J Nutr.

[10] Bonder MJ, Kurilshikov A, Tigchelaar EF, Mujagic Z, Imhann F, Vila AV, Deelen P, Vatanen T, Schirmer M, Smeekens SP (2016). The effect of host genetics on the gut microbiome. Nat Genet.

[11] Egerton S, Culloty S, Whooley J, Stanton C, Ross RP (2018). The gut microbiota of marine fish. Front Microbiol.

[12] Mohajeri MH, La Fata G, Steinert RE, Weber P (2018). Relationship between the gut microbiome and brain function. Nutr Rev.

[13] Picca A, Fanelli F, Calvani R, Mule G, Pesce V, Sisto A, Pantanelli C, Bernabei R, Landi F, Marzetti E (2018). Gut Dysbiosis and muscle aging: searching for novel targets against sarcopenia. Mediat Inflamm.

[14] Grosicki GJ, Fielding RA, Lustgarten MS (2018). Gut microbiota contribute to age-related changes in skeletal muscle size, composition, and function: biological basis for a gut-muscle Axis. Calcif Tissue Int.

[15] Dugas LR, Lie L, Plange-Rhule J, Bedu-Addo K, Bovet P, Lambert EV, Forrester TE, Luke A, Gilbert JA, Layden BT (2018). Gut microbiota, short chain fatty acids, and obesity across the epidemiologic transition: the METS-microbiome study protocol. BMC Public Health.

[16] Hernández MAG, Canfora EE, Jocken JWE, Blaak EE. The Short-Chain Fatty Acid Acetate in Body Weight Control and Insulin Sensitivity. Nutrients. 2019 Aug 18;11(8):1943. 10.3390/nu11081943. 10.3390/nu11081943 PMC6723943 31426593

[17] Salem M, Kenney PB, Rexroad CE, Yao J (2006). Molecular characterization of muscle atrophy and proteolysis associated with spawning in rainbow trout. Comp Biochem Physiol Part D Genomics Proteomics.

[18] Argiles JM, Campos N, Lopez-Pedrosa JM, Rueda R, Rodriguez-Manas L (2016). Skeletal muscle regulates metabolism via Interorgan crosstalk: roles in health and disease. J Am Med Dir Assoc.

[19] Nagaraju K (2001). Immunological capabilities of skeletal muscle cells. Acta Physiol Scand.

[20] Magnoni LJ, Roher N, Crespo D, Krasnov A, Planas JV. In Vivo Molecular Responses of Fast and Slow Muscle Fibers to Lipopolysaccharide in a Teleost Fish, the Rainbow Trout (*Oncorhynchus mykiss*). Biology (Basel). 2015;4(1):67–87. 10.3390/biology4010067 PMC4381218 25658438

[21] Lahiri S, Kim H, Garcia-Perez I, Reza MM, Martin KA, Kundu P, Cox LM, Selkrig J, Posma JM, Zhang H, Padmanabhan P, Moret C, Gulyás B, Blaser MJ, Auwerx J, Holmes E, Nicholson J, Wahli W, Pettersson S. The gut microbiota influences skeletal muscle mass and function in mice. Sci Transl Med. 2019;11(502). 10.1126/scitranslmed.aan5662 PMC7501733 31341063

[22] Shanahan F, van Sinderen D, O'Toole PW, Stanton C (2017). Feeding the microbiota: transducer of nutrient signals for the host. Gut.

[23] Kokou F, Sasson G, Nitzan T, Doron-Faigenboim A, Harpaz S, Cnaani A, Mizrahi I: Host genetic selection for cold tolerance shapes microbiome composition and modulates its response to temperature. Elife. 2018;7. 10.7554/eLife.36398 PMC6277203 30454554

[24] Smith CC, Snowberg LK, Gregory Caporaso J, Knight R, Bolnick DI (2015). Dietary input of microbes and host genetic variation shape among-population differences in stickleback gut microbiota. ISME J.

[25] Chapagain P, Arivett B, Cleveland BM, Walker DM, Salem M. Analysis of the fecal microbiota of fast- and slow-growing rainbow trout (*Oncorhynchus mykiss*). BMC Genomics. 2019;20(1):788. 10.1186/s12864-019-6175-2 PMC6819385 31664902

[26] Wexler HM (2007). Bacteroides: the good, the bad, and the nitty-gritty. Clin Microbiol Rev.

[27] Brennan CA, Garrett WS. *Fusobacterium nucleatum* - symbiont, opportunist and oncobacterium. Nat Rev Microbiol. 2019;17(3):156–66. 10.1038/s41579-018-0129-6 PMC6589823 30546113

[28] Mayhew JW, Onderdonk AB, Gorbach SL. Effects of time and growth media on short-chain fatty acid production by *Bacteroides fragilis*. Appl Microbiol. 1975;29(4):472–5. 10.1128/am.29.4.472-475.1975 PMC187008 1124920

[29] Tuner K, Baron EJ, Summanen P, Finegold SM. Cellular fatty acids in *Fusobacterium* species as a tool for identification. J Clin Microbiol. 1992;30(12):3225–9. 10.1128/jcm.30.12.3225-3229.1992 PMC270637 1452706

[30] Michl SC, Ratten JM, Beyer M, Hasler M, LaRoche J, Schulz C. The malleable gut microbiome of juvenile rainbow trout (*Oncorhynchus mykiss*): diet-dependent shifts of bacterial community structures. PLoS One. 2017;12(5):e0177735. 10.1371/journal.pone.0177735 PMC5428975 28498878

[31] Navarrete P, Magne F, Araneda C, Fuentes P, Barros L, Opazo R, Espejo R, Romero J. PCR-TTGE analysis of 16S rRNA from rainbow trout (*Oncorhynchus mykiss*) gut microbiota reveals host-specific communities of active bacteria. PLoS One. 2012;7(2):e31335. 10.1371/journal.pone.0031335 PMC3290605 22393360

[32] Larsen AM, Mohammed HH, Arias CR (2014). Characterization of the gut microbiota of three commercially valuable warmwater fish species. J Appl Microbiol.

[33] Roeselers G, Mittge EK, Stephens WZ, Parichy DM, Cavanaugh CM, Guillemin K, Rawls JF (2011). Evidence for a core gut microbiota in the zebrafish. ISME J.

[34] Zhang YM, Wong TY, Chen LY, Lin CS, Liu JK. Induction of a futile Embden-Meyerhof-Parnas pathway in *Deinococcus radiodurans* by Mn: possible role of the pentose phosphate pathway in cell survival. Appl Environ Microbiol. 2000;66(1):105–12. 10.1128/aem.66.1.105-112.2000 PMC91792 10618210

[35] Narrowe AB, Albuthi-Lantz M, Smith EP, Bower KJ, Roane TM, Vajda AM, Miller CS (2015). Perturbation and restoration of the fathead minnow gut microbiome after low-level triclosan exposure. Microbiome.

[36] Shen H, Zang Y, Song K, Ma Y, Dai T, Serwadda A. A meta-transcriptomics survey reveals changes in the microbiota of the Chinese mitten crab *Eriocheir sinensis* infected with Hepatopancreatic necrosis disease. Front Microbiol. 2017;8:732. 10.3389/fmicb.2017.00732 PMC5405120 28491058

[37] Sakai M, Yoshida T, Astuta S, Kobayashi M (1995). Enhancement of resistance to vibriosis in rainbow trout, O*ncorhynchus mykiss* (Walbaum) by oral administration of C*lostridium butyricum* bacteria. J Fish Dis.

[38] Taoka Y, Maeda H, Jo J-Y, Jeon M-J, Bai SC, Lee W-J, Yuge K, Koshio S (2006). Growth, stress tolerance and non-specific immune response of Japanese flounder *Paralichthys olivaceus* to probiotics in a closed recirculating system. Fisheries Science.

[39] Nikitin DI, Strompl C, Oranskaya MS, Abraham WR. Phylogeny of the ring-forming bacterium *Arcicella aquatica* gen. nov., sp. nov. (ex Nikitin et al. 1994), From a freshwater neuston biofilm. Int J Syst Evol Microbiol. 2004;54(Pt 3):681–4. 10.1099/ijs.0.02896-0 15143007

[40] Wang C, Sun G, Li S, Li X, Liu Y (2018). Intestinal microbiota of healthy and unhealthy Atlantic salmon *Salmo salar* L. in a recirculating aquaculture system. J Oceanol Limnol.

[41] Penders J, Thijs C, Vink C, Stelma FF, Snijders B, Kummeling I, van den Brandt PA, Stobberingh EE (2006). Factors influencing the composition of the intestinal microbiota in early infancy. Pediatrics.

[42] Tap J, Mondot S, Levenez F, Pelletier E, Caron C, Furet JP, Ugarte E, Munoz-Tamayo R, Paslier DL, Nalin R (2009). Towards the human intestinal microbiota phylogenetic core. Environ Microbiol.

[43] Wang J, Lang T, Shen J, Dai J, Tian L, Wang X (2019). Core gut Bacteria analysis of healthy mice. Front Microbiol.

[44] Sugita H, Miyajima C, Deguchi Y. The vitamin B12-producing ability of intestinal bacteria isolated from tilapia and channel catfish. Nippon Suisan Gakkaishi. 1989;4:701.

[45] Frampton J, Murphy KG, Frost G, Chambers ES (2020). Short-chain fatty acids as potential regulators of skeletal muscle metabolism and function. Nat Metab.

[46] Hyun DW, Shin NR, Kim MS, Kim JY, Kim PS, Oh SJ, Whon TW, Bae JW. *Cloacibacterium haliotis* sp. nov., isolated from the gut of an abalone, Haliotis discus hannai. Int J Syst Evol Microbiol. 2014;64(Pt 1):72–7. 10.1099/ijs.0.054585-0 24014625

[47] Tyagi A, Singh B, Billekallu Thammegowda NK, Singh NK (2019). Shotgun metagenomics offers novel insights into taxonomic compositions, metabolic pathways and antibiotic resistance genes in fish gut microbiome. Arch Microbiol.

[48] Llewellyn MS, McGinnity P, Dionne M, Letourneau J, Thonier F, Carvalho GR, Creer S, Derome N. The biogeography of the Atlantic salmon (*Salmo salar*) gut microbiome. ISME J. 2016;10(5):1280–4. 10.1038/ismej.2015.189 PMC5029221 26517698

[49] Kirchhoff H, PB, fischer M, Flossdorf J, Heitmann J, Khattab B, Lopatta D, Rosengarten R, GS, Yousef C. *Mycoplasma mobile* sp. nov., a New Species from Fish. Int J Syst Bacteriol. 1987;37:192–7.

[50] Schwiertz A, Taras D, Schafer K, Beijer S, Bos NA, Donus C, Hardt PD (2010). Microbiota and SCFA in lean and overweight healthy subjects. Obesity (Silver Spring).

[51] Ali A, Al-Tobasei R, Kenney B, Leeds TD, Salem M (2018). Integrated analysis of lncRNA and mRNA expression in rainbow trout families showing variation in muscle growth and fillet quality traits. Sci Rep.

[52] Lustgarten MS (2019). The role of the gut microbiome on skeletal muscle mass and physical function: 2019 update. Front Physiol.

[53] Hu J, Lin S, Zheng B, Cheung PCK (2018). Short-chain fatty acids in control of energy metabolism. Crit Rev Food Sci Nutr.

[54] Bui TP, Ritari J, Boeren S, de Waard P, Plugge CM, de Vos WM. Production of butyrate from lysine and the amadori product fructoselysine by a human gut commensal. Nat Commun. 2015;6:10062. 10.1038/ncomms10062 PMC4697335 26620920

[55] Barker HA, Kahn JM, Hedrick L. Pathway of lysine degradation in *Fusobacterium nucleatum*. J Bacteriol. 1982;152(1):201–7. 10.1128/jb.152.1.201-207.1982 PMC221392 6811551

[56] Cabrera-Mulero A, Tinahones A, Bandera B, Moreno-Indias I, Macias-Gonzalez M, Tinahones FJ (2019). Keto microbiota: a powerful contributor to host disease recovery. Rev Endocr Metab Disord.

[57] Evans M, Cogan KE, Egan B (2017). Metabolism of ketone bodies during exercise and training: physiological basis for exogenous supplementation. J Physiol.

[58] McGlory C, Calder PC, Nunes EA (2019). The influence of Omega-3 fatty acids on skeletal muscle protein turnover in health, disuse, and disease. Front Nutr.

[59] Tallima H, El Ridi R (2018). Arachidonic acid: physiological roles and potential health benefits - a review. J Adv Res.

[60] Tilocca B, Burbach K, Heyer CME, Hoelzle LE, Mosenthin R, Stefanski V, Camarinha-Silva A, Seifert J (2017). Dietary changes in nutritional studies shape the structural and functional composition of the pigs' fecal microbiome-from days to weeks. Microbiome.

[61] Leskawa KC, Erwin RE, Buse PE, Hogan EL (1988). Glycosphingolipid biosynthesis during myogenesis of rat L6 cells in vitro. Mol Cell Biochem.

[62] Papini N, Anastasia L, Tringali C, Dileo L, Carubelli I, Sampaolesi M, Monti E, Tettamanti G, Venerando B (2012). MmNEU3 sialidase over-expression in C2C12 myoblasts delays differentiation and induces hypertrophic myotube formation. J Cell Biochem.

[63] Lipina C, Hundal HS (2017). Lipid modulation of skeletal muscle mass and function. J Cachexia Sarcopenia Muscle.

[64] Qi Y, Jiang C, Cheng J, Krausz KW, Li T, Ferrell JM, Gonzalez FJ, Chiang JY (2015). Bile acid signaling in lipid metabolism: metabolomic and lipidomic analysis of lipid and bile acid markers linked to anti-obesity and anti-diabetes in mice. Biochim Biophys Acta.

[65] Vítek L, Haluzík M (2016). The role of bile acids in metabolic regulation. J Endocrinol.

[66] Kozich JJ, Westcott SL, Baxter NT, Highlander SK, Schloss PD (2013). Development of a dual-index sequencing strategy and curation pipeline for analyzing amplicon sequence data on the MiSeq Illumina sequencing platform. Appl Environ Microbiol.

[67] ILLUMINA: Miseq System Guide. In*.*, vol. Document # 1000000061014 v00 2018.

[68] Schloss PD, Westcott SL, Ryabin T, Hall JR, Hartmann M, Hollister EB, Lesniewski RA, Oakley BB, Parks DH, Robinson CJ, Sahl JW. Introducing mothur: open-source, platform-independent, community-supported software for describing and comparing microbial communities. Appl Environ Microbiol. 2009;75(23):7537–41. 10.1128/AEM.01541-09 PMC2786419 19801464

[69] Kowallik V, Miller E, Greig D. The interaction of S*accharomyces paradoxus* with its natural competitors on oak bark. Mol Ecol. 2015;24(7):1596–610. 10.1111/mec.13120 PMC4405091 25706044

[70] Carlsen T, Aas AB, Lindner D, Vrålstad T, Schumacher T, Kauserud H. Don’t make a mista(g)ke: is tag switching an overlooked source of error in amplicon pyrosequencing studies? Fungal Ecol. 2012;5:747–9.

[71] Quast C, Pruesse E, Yilmaz P, Gerken J, Schweer T, Yarza P, Peplies J, Glöckner FO. The SILVA ribosomal RNA gene database project: improved data processing and web-based tools. Nucleic Acids Res. 2012;41:D590–6. 10.1093/nar/gks1219 PMC3531112 23193283

[72] Rognes T, Flouri T, Nichols B, Quince C, Mahe F (2016). VSEARCH: a versatile open source tool for metagenomics. PeerJ.

[73] Lindahl BD, Nilsson RH, Tedersoo L, Abarenkov K, Carlsen T, Kjøller R, Kõljalg U, Pennanen T, Rosendahl S, Stenlid J, Kauserud H. Fungal community analysis by high-throughput sequencing of amplified markers--a user's guide. New Phytol. 2013;199(1):288–99. 10.1111/nph.12243 PMC3712477 23534863

[74] Walker DM, Leys JE, Grisnik M, Grajal-Puche A, Murray CM, Allender MC (2019). Variability in snake skin microbial assemblages across spatial scales and disease states. ISME J.

[75] Oksanen J, Blanchet FG, Kindt R, Legendre P, Minchin PR, O’hara RB, Simpson GL, Solymos P, Stevens MH, Wagner H: vegan: community ecology package. R package version 2.5–2. In.; 2018: https://CRAN.R-project.org/package=vegan.

[76] H. W: The split-apply-combine strategy for data analysis. J Stat Softw. In*.*; 2011: 1–29. http://www.jstatsoft.org/v40/i01/URL.

[77] Wickham H FR, Henry L, Müller K: dplyr: A grammar of data manipulation. R package version 0.7.6. . In.; 2018: https://CRAN.R-project.org/package=dplyr.

[78] Hadley Wickham, Winston Chang, Lionel Henry, Thomas Lin Pedersen, Kohske Takahashi, Claus Wilke, Kara Woo, Hiroaki Yutani, Dunnington D: ggplot2: Create elegant data visualisations using the grammar of graphics, R Package version 3.3.0: https://cran.r-project.org/web/packages/ggplot2. 2020.

[79] Brockhoff B. lmerTest v2.0-36 https://www.rdocumentation.org/packages/lmerTest.

[80] Barton K: Multi-Model Inference:R package version 1.43.15,. In*.*: https://cran.r-project.org/package=MuMIn; 2019-12-19.

[81] Douglas Bates, Martin Maechler, Ben Bolker, Steven Walker, Rune Haubo Bojesen Christensen, Henrik Singmann, Bin Dai, Fabian Scheipl, Gabor Grothendieck, Peter Green et al: Package lme4. R package version 1.1–21 . https://cran.r-project.org/web/packages/lme4/index.html. 2019.

[82] Asshauer KP, Wemheuer B, Daniel R, Meinicke P (2015). Tax4Fun: predicting functional profiles from metagenomic 16S rRNA data. Bioinformatics.

[83] Love MI, Huber W, Anders S (2014). Moderated estimation of fold change and dispersion for RNA-seq data with DESeq2. Genome Biol.

[84] Mangiafico S: rcompanion: Functions to support extension education program evaluation, R package version 2.0.10. https://CRAN.R-project.org/package=rcompanion. 2019.

[85] Murrell P (2005). R graphics.

[86] Wickham H: Tidyverse: R package version 1.3.0. In*.*: https://cran.r-project.org/package=tidyverse; 2019-11-21..

[87] Bates, D, M, M, B, B, al. e: Package lme4. R package version 1.1–7. https://cran.r-project.org/web/packages/lme4/index.html. 2014.

[88] Oksanen, J, B, FG, F, M, al. e: Vegan: Community Ecology Package. R package version 2.5–2. 2018.

[89] Wickham H. ggplot2: Elegant graphics for dData analysis. New York: Springer-Verlag; 2016.

[90] Kold R (2015). pheatmap: pretty heatmaps R package version 1.0.8.

