# Supplementary material for: Distinct microbial assemblages associated with genetic selection for high- and low- muscle yield in rainbow trout
Source: BMC Genomics. 2020 Nov 23;21:820. doi: 10.1186/s12864-020-07204-7 (PMC7684950; doi:10.1186/s12864-020-07204-7)
Supplement: Supplementary file 5 — Additional file 5. Comparison of gut and environmental samples. [file 12864_2020_7204_MOESM5_ESM.docx]

**Comparison of microbial turnover in the gut and environment**

A total of 2,256 OTUs were identified from all feces, feed and water samples. To determine if the gut microbiota differ from that of the environment (water or feed), Bray-Curtis distance matrices were compared and the results indicated no significant differences of the feed and environment microbial assemblages with feces microbiota (F_1,40=_ 1.15 and p>0.05, R^2^=2.8%). In addition, the microbiota among ARS-FY-H and ARS-FY-L genetic lines, feed, and water were compared based on Bray-Curtis dissimilarity matrices. The nMDS ordination showed that feed and water samples were clustered together with most of the Low-muscle yield genetic line samples. However, a pairwise Adonis test indicates no significant differences between the 2 genetic lines (ran individually) with feed or water samples (p>0.05) (Additional figure. 1). This might be because fish acquire microbiota from feed and, from water in which they are reared. Similarly,


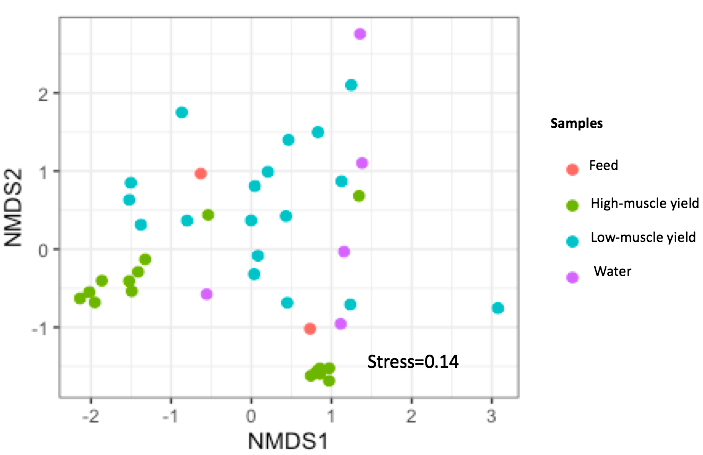


**Figure 1**. Beta diversity patterns of High-, Low-muscle yield genetic lines microbiota compared to feed and water; data was visualized under nMDS ordination. (stress=0.14)
